# Preliminary Results of Instantaneous Centre of Rotation Change after Thoracic Instrumentation in Adolescent Idiopathic Scoliosis Patients

**DOI:** 10.5704/MOJ.2211.018

**Published:** 2022-11

**Authors:** A Asma, C Onal, ON Eroglu, RH Berk

**Affiliations:** 1 Department of Orthopaedics, Nemours Children's Hospital Delaware, Wilmington, United States; 2 Department of Radiology, Kars Harakani State Hospital, Kars, Turkey; 3 Department of Orthopaedics, Dokuz Eylul University, Izmir, Turkey

**Keywords:** instantaneous centre of rotation, adolescent idiopathic scoliosis, sagittal balance, cervical balance

## Abstract

**Introduction:**

The instantaneous centre of rotation (ICR) is the centre of motion with zero velocity where a rigid body moves in a particular plane. ICR, as a dynamic measurement, gives more accurate results in terms of quality and quantity of the vertebral motions compared to range of motion (ROM). We aimed to determine the effect of thoracic instrumentation on cervical movement of adolescent idiopathic scoliosis (AIS) patients who had undergone thoracic level instrumentation by measuring pre-operative and post-operative ICR change in a pilot study

**Materials and methods:**

A total of 25 AIS patients were included in this study. C4-C5 and C6-C7 segmental ICR were determined by pre-operative and post-operative cervical flexion-extension radiographs. In addition, cervical sagittal parameters and global sagittal parameters were investigated.

**Results:**

There was no statistically significant change in ICR location post-operatively in both x and y coordinates at C4-C5 segment (p: 0.326 and p: 0.946, respectively) and C6-C7 segment (p: 0.209, p: 0.086, respectively). There was a positive correlation between LCL and C4-C5 ICR y coordinate (r: 0.481), but not with C6-C7 ICR y coordinate (r: -0.2, p: 0.398). T5-T12 kyphosis decreased (p: 0.002) and T1 pelvic angle (0.003), SVA (0.02) and sacral slope (0.049) increased significantly post-operatively. T1S was correlated with LCL (r: 0.595, p: 0.002), T5-T12 kyphosis (r: 0.423, p: 0.035), SVA (r: 0.658, p<0.001) and C2-C7 SVA (r: 0.416, p: 0.039).

**Conclusion:**

The ICR for cervical region was not changed post-operatively in AIS patients with thoracic instrumentation. There was no relationship found between the development of post-operative cervical kyphosis or lordosis and ICR, which represents the quality and quantity of intervertebral motion. The T1 vertebra plays a key role for cervical, thoracic, and global parameters interaction.

## Introduction

The instantaneous centre of rotation (ICR) is the centre of motion with zero velocity where a rigid body moves in a particular plane^1^. It is shown that ICR gives more accurate results of intervertebral segmental movement quality compared to the range of motion (ROM)^2^. ICR can detect not only normal movements but also abnormal movements, which makes it more useful for determining the quality of motion on a specific segment^3^. ICR moves anterior and superior in the presence of cervical degenerative changes, where superior movement of ICR is caused by decrease in disc height and anterior movement of ICR is caused by anterior osteophytes^2^.

The compressive, shearing force vectors can change the ICR position on cervical spine^4,5^. Amevo *et al* evaluated 109 patients with neck pain and found that 46% of patients had abnormal ICR, whereas 26% had marginal ICR^6^. Mayer *et al* compared 12 patients with cervicogenic headache to a control group consisted of 18 healthy individuals, and reported that patients who had cervicogenic headache, had abnormally located ICR in C2-C3 segment, and this was normalised by therapeutic injection of C2 nerve root afterwards^7^. In another study, anterior longitudinal ligament, annulus fibrosus, and uncovertebral joints were destructed, respectively in cadaver models, and consequently ICR moved posteriorly to healthier segments after serial sectioning of anterior structures^8^. Haher *et al* showed in the simulated vertebral fracture model, ICR located more posteriorly and inferiorly in the anterior and middle column simultaneous fractures^9^.

There is an increased attention to understand the cervical sagittal alignment in adolescent idiopathic scoliosis (AIS) patients^10-13^. There is a mutual relationship between the cervical spinal balance (CSB) and global spinal balance (GSB) alterations. An increase in pelvic incidence (PI) can cause an increase in lumbar lordosis, which leads to an increase in thoracic kyphosis and cervical lordosis^14^. On the other hand, the decrease in lumbar lordosis causes the pelvic retroversion and positive sagittal vertical axis (SVA), which eventually breeds an increase in cervical lordosis to maintain horizontal gaze. If spinal deformity originates from the cervical region as in the cervical kyphosis, pelvic retroversion increases to provide enough pelvic tilt for the maintenance of horizontal gaze. However, those reports were based on single time static image of the lateral cervical spine and whole spine. In this study, we aimed to joint dynamic quality and quantity of measurement tool “ICR” with sagittal plane analysis, and we evaluated both cervical and global sagittal plane concomitantly. The aim of this pilot study is to determine the effect of thoracic instrumentation on cervical kinematic by comparing pre-operative and post-operative location of ICR. We hypothesise that the ICR location will change after thoracic instrumentation in AIS patients.

## Materials and Methods

The pilot study approval obtained from the local hospital ethical committee. The inclusion criterion was AIS diagnosis with major curve more than 45° and exclusion criteria were as follows, history of congenital scoliosis, neuromuscular scoliosis, previous spinal surgery, the presence of cervical fusion, and poor radiographic images. After IRB approval, all the patients who have enrolled for posterior spinal fusion in limits of inclusion and exclusion criteria between June 2017- August 2018 were recruited for the study. Posteroanterior (PA) and lateral full spine radiograph, cervical radiographs in neutral, full flexion and in full extension positions were all obtained. Cervical neutral images with standing erect posture were acquired initially with radiograph beam focused on C4 vertebra, while the tube-cassette distance was set to 150cm. Then, patients were asked to do cervical flexion and extension as much as they can do, and radiograph images were acquired in these postures again. The patients were followed post-operatively at the outpatient clinic, and the images were repeated after patients’ well-being, independent movement, painless neck movement were established at six-week post-operative control. The data collection was done in prospective manner and analysis done retrospectively.

**Fig. 1.**
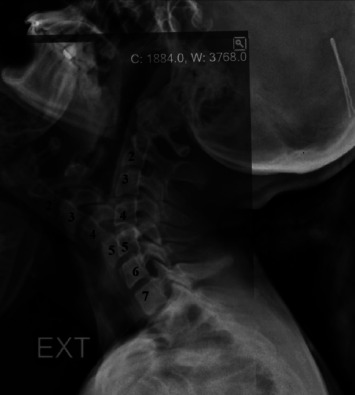
The overlap of C7 vertebra at cervical flexion and extension images for C6-C7 segment.

**Fig. 2.**
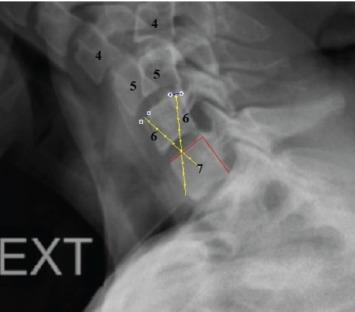
The ICR detection for the C6-C7 segment of the patient in the Fig 1. The ICR was localised at the intersection of the vertices from the midpoint of the two lines. The two lines were formed by the four dots on the anterior and posterior corner of C6 vertebrae in flexion and extension radiographs.

The primary outcome variable was ICR location change. There are numerous techniques described to calculate ICR for vertebral segments^15-18^. In accordance with improvement in image processing software, we conducted our measurement protocol via two software programs. We detected ICR for C4-C5 segment, which was the main mobile sub-axial cervical segments and for the C6-C7 segment, which was the closest cervical segment to the thoracic region. C5 vertebra for C4-C5 segment and C7 vertebra for C6-C7 segment were overlapped in flexion and extension views on licensed Adobe Photoshop CS6 [California, US, 2012] with three-point overlapping technique (Fig. 1). Then these images were uploaded to the licensed AutoCAD [California, US, 2007] software to make measurements as previously described in literature^1,4,17^. Two points of the upper vertebra in flexion and similar points in extension views were chosen, then a straight line to merge these points with their correlates was drawn. After having two straight lines, the midpoints were intersected, and this would define ICR of the associated segment (Fig. 2).

**Fig. 3.**
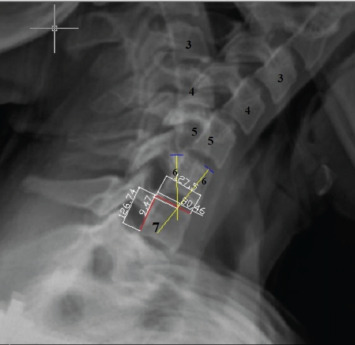
The ICR in segment C6-7. The yellow lines indicate the vertices drawn from the midpoints of the four points on the anterior and posterior corner of the C6 vertebrae in the overlapped flexion and extension film, the red lines indicate the superior (x-axis) and posterior (y-axis) walls of the C7 vertebra, and the white lines indicates the perpendicular lines from the ICR centre to the x and y-axis. The coordinates of ICR in x-axis and y-axis was normalised to each vertebral border height. In this example x coordinate was 0.63(80.46/127.5), and y coordinate was 0,074 (9.47/126.74).

The same procedure was repeated at follow-up controls after a free and painless mobilisation period obtained at postoperative six weeks. A coordinate system was established to express the determined location of the ICR as in literature^2^. The x-y axis originated from the postero-superior corner of the lower vertebra of the associated segment. Depending upon this, the x-axis was drawn from the postero-superior corner of the lower vertebra through an anterior direction along the superior border, and the y-axis was drawn from the same origin through an inferior direction along the posterior border of the vertebra. The coordinates (x, y) of ICR were normalised as percentages based on the width and height of the lower vertebral body for offsetting individual variations in the sizes of the vertebrae (Fig. 3).

We also looked for upper instrumentation level (being T2 or not), C0-C2 upper cervical lordosis (UCL), C2-C7 lower cervical lordosis (LCL), T1 slope(T1S), C7 sagittal vertical axis (SVA), C2-C7 SVA, T1 pelvic angle (TPA), PI, pelvic tilt (PT), sacral slope (SS), T1-T5 kyphosis, T5-T12 kyphosis as secondary outcome variables.

All the patient's data were analysed in SPSS 22.0 program [Chicago, U.S 2014]. After checking the normal distribution of our cohort by Kolmogorov Smirnov test, Wilcoxon signed rank tests were used to analyse pre-operative and postoperative values for non-parametric parameters. Spearman correlation test was used to check the correlation between ICR change and the other parameters described above. Pearson chi-square analysis was used to check the relation for T2 instrumentation. P value is established as 0,05. All parameters were measured after two weeks of primary measure to test intra-observer error. Intra-observer correlation (ICC) was found to be 0.715, which indicates a strong agreement. A power analysis with minimum detectable effect of 0.1 for ICR change on x-axis found out 67% power.

## Results

A total of 25 patients were enrolled in this pilot study. There were 10 patients with Lenke type 1, six patients with Lenke type 2, four patients with Lenke type 3, three patients with Lenke type 4 and two patients with Lenke 6. The thirteen of the patients were instrumented up to T2, and rest of it instrumented below T2. There was no statistically significant change in ICR location post-operatively in both x and y coordinates at C4-C5 segment (p: 0.326 and p: 0.946, respectively) and C6-C7 segments (p: 0.209, p: 0.086, respectively). The T5-T12 kyphosis decreased postoperatively (p: 0,002). The TPA (p: 0,003), SS (p: 0,049) and SVA (p: 0.02) increased post-operatively (positive change in global sagittal balance). There was no statistically significant change UCL, LCL, T1S, T1-T5K, C2-C7 SVA postoperatively (Table I).

**Table I:** Pre-operative – post-operative comparison of ICR location and sagittal plane parameters

	Mean	Std. Deviation	Sig. (2-tailed)
Pre-op C4-C5 ICR x-axis	0.51	0.12	0.326
Post-op C4-C5 ICR x-axis	0.47	0.17	
Pre-op C4-C5 ICR y-axis	0.48	0.17	0.946
Post-op C4-C5 ICR y-axis	0.50	0.24	
Pre-op C6-C7 ICR x-axis	0.53	0.17	0.129
Post-op C6-C7 ICR x-axis	0.43	0.08	
Pre-op C6-C7 ICR y-axis	0.71	1.06	0.464
Post-op C6-C7 ICR y-axis	0.95	0.89	
Pre-op UCL	35.44	10.28	0.563
Post-op UCL	36.60	10.46	
Pre-op LCL	6.50	14.55	0.353
Post-op LCL	9.22	16.52	
Pre-op T5-T12 Kyphosis	32.14	14.23	0.002
Post-op T5-T12 Kyphosis	21.13	10.23	
Pre-op T1-T5 Kyphosis	13.80	14.62	0.545
Post-op T1- T5 Kyphosis	18.20	12.16	
Pre-op T1 Slope	18.69	11.30	0.819
Post-op T1 Slope	18.80	12.75	
Pre-op T1 Pelvic angle	7.33	9.15	0.003
Post-op T1 Pelvic angle	11.08	9.75	
Pre-op SS	31.85	10.05	0.049
Post-op SS	35.57	12.51	
Pre-op PT	16.12	9.61	0.128
Post-op PT	13.53	8.67	
Pre-op C2- C7 SVA	19.40	14.29	0.174
Post-op C2-C7 SVA	16.92	11.92	
Pre-op SVA	-35.13	42.56	0.02
Post-op SVA	-1.68	51.91	

Abbreviations - ICR: Instantaneous centre of rotation, UCL: Upper cervical lordosis (C0-C2), LCL: Lower cervical lordosis (C2-C7), SS: Sacral slope, PT: Pelvic tilt, SVA: Sagittal vertical axis

There was positive correlation between C4-C5 segment ICR y coordinate and LCL. (r: 0.481, p: 0.015). Twelve of the patients have LCL decreased accompanied by superior movement in ICR centre where eight of the patients have LCL increased accompanied by inferior movement in ICR centre. An inferior movement in ICR reflected the decreased ROM in associated segments and notably this occurs with an increase in LCL post-operatively. As we know ICR refers to quality and ROM of the associated segment, it is notable to find a decrease in ROM correlated with an increase in LCL. Hence, there was no other correlation between C4-C5 segment ICR y coordinate and other sagittal plane parameters including UCL, T1 slope and T1-T5 kyphosis, this result should be interpreted as with an increase in LCL, there is a decrease in segmental movement of C4-C5 segment. One should remember that this is only for C4-C5 segment change. We also checked for ICR of C6-C7 segment. After overlapping of flexion and extension view of the cervical region, we found out that there was no movement in C6-C7 segment in five patients (the cervical movement was done by upper cervical segments in these patients). As a rule, ICR cannot be calculated if the segmental motion is less than 5° in the sagittal plane^6^. Postoperatively, nine more patients lost their movement in C6-C7 segment, and five patients had also more inferiorly localised ICR, which indicates decreased ROM in this region. Totally, fourteen patients had an inferior movement on ICR C6-C7 y-axis. Even though our result was not statistically significant (p: 0.086), there was a tendency to decrease in C6-C7 segment movement after thoracic instrumentation. Besides this, there was no correlation detected between C6-C7 segment y coordinate and LCL (r: -0.2, p: 0.398). This might be interpreted as there was no relation between ROM of a spine region and its lordotic or kyphotic view on radiograph image, which was taken in a static moment. In another point of view, post-operative increase in cervical lordosis would not indicate an increase in cervical ROM and movement quality, just as kyphotic cervical spine would not indicate a decrease in quality and quantity of cervical spine motion.

**Fig. 4.**
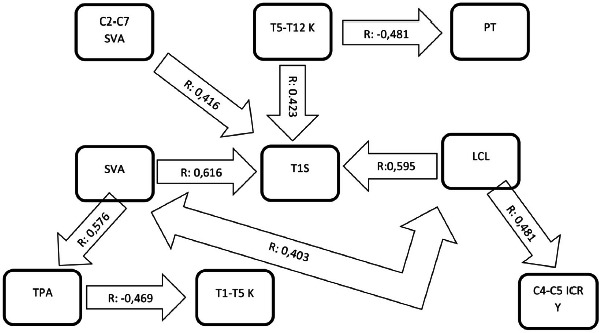
Spearman correlation analysis of variables. Note that the T1 slope acts as a keystone. T1S: T1 slope, LCL: Lower cervical lordosis, ICR: Instantaneous center of rotation, PT: Pelvic tilt, SVA: Sagittal vertical axis, TPA: T1 Pelvic angle, T1-T5 K: T1-T5 Kyphosis angle

There was a strong correlation between LCL and T1S even significance value was accepted as 0.01. In addition, an increase in T1S was correlated with an increase in LCL to maintain horizontal gaze. Another point to be emphasised is the compensation of increased T1 tilt primarily was done through the most adjacent segment rather than the upper cervical region. LCL was also correlated with SVA, where increased lordosis puts C7 vertebra more anteriorly and results in positive SVA in the sagittal alignment (Fig. 4).

T1S and T5-T12K were correlated with each other. A postoperative increase in T5-T12 kyphosis occurred simultaneously with an increase in T1S. However, there was no correlation between T1-T5K and T1S. We related this result to the fact that T1S was affected more by a larger thoracic region. Moreover, most of the patients were not instrumented at T1 and a segmental kyphosis at T1-T2 segment could make T1 vertebra tilted post-operatively. T1S was also correlated with SVA. A more anteriorly placed C7 vertebra caused by increased cervical lordosis, and increased T1 slope moved the sagittal balance into positive direction. C2-C7 SVA was also similarly correlated with T1S. There was negative correlation between TPA and T1-T5K. This result might be related to compensation of positive sagittal balance through T1-T5 region to maintain balance and horizontal gaze (Table II).

**Table II:** Spearman correlation results of the significant variables

Spearman Correlation	LCL	T5-T12 kyphosis	T1-T5 kyphosis	SVA	C2-C7 SVA
ICR y-axis	r: 0.481				
	p: 0.015				
T1 slope	r: 0.595	r: 0.423		r: 0.658	r: 0.416
	p: 0.002	p: 0.035		p<0.001	p: 0.039
PT		r: -0.481			
		p: 0.015			
TPA			r: -0.469	r: 0.576	
			p: 0.018	p: 0.003	
LCL				r: 0.403	
				p: 0.046	

Abbreviations - T1S: T1 slope, LCL: Lower cervical lordosis, ICR: Instantaneous centre of rotation, PT: Pelvic tilt, SVA: Sagittal vertical axis, TPA: T1 Pelvic angle, T1-T5 K: T1-T5 Kyphosis angle

We additionally classified our patients into two groups: patients with pre-operative T5-T12 kyphosis ≥20° and <20°. Chi-square analysis revealed that all the patients with increased LCL had pre-operative T5-T12 kyphosis <20° (p: 0.028), and 10 of the 19 patients with pre-operative T5-T12 kyphosis more than 20° had decreased LCL post-operatively. The results showed that post-operative cervical lordosis might decrease after thoracic instrumentation especially in patients with more than 20° pre-operative T5-T12 thoracic kyphosis. When patients grouped according to whether upper instrumented level (UIL) was T2 or not, there was a strong relationship with LCL, as LCL decreased in patients with T2 UIL, and increased in patients with instrumented below T2 (p: 0.002) (Table III).

**Table III:** T2 instrumentation and T5-T12 kyphosis relationship with LCL change

		LCL increased		LCL decreased				Sig (2-tailed)
		Count	Row N %	Column N %	Count	Row N %	Column N %	
T2 instrumented	Yes	4	30.8	26.7	9	69.2	90	0.003
	No	11	91.7	73.3	1	8.3	10	
Pre-op T5-T12 Kyphosis <20°	Yes	6	100	40	0	0	0	0.028
	No	9	47	60	10	52.6	100	
Total		15	100	100	10	100	100	

Abbreviation - LCL: Lower Cervical Lordosis

## Discussion

We can detect abnormalities only in one plane by using ROM, while ICR can evaluate the problems in multiple planes. The ICR could also give more information to evaluate the quality of motion and to identify the reason for any decrease in motion rather than a quantitative evaluation as in ROM measurements. To our knowledge, this was the first study that was conducted on the AIS population for post-operative ICR change. First, we showed that cervical segments’ ICR locations were not affected by thoracic instrumentation. This can be interpreted as thoracic instrumentation may alter the sagittal spinal curvatures, but overall, the kinematics of sub-axial cervical region were not changing.

Hwang *et al*^19^ reported that at least 60° ROM was required to show any abnormality after soft tissue injury, however ICR determination unveils soft tissue injury without need of ROM obligation. Schmidt *et al*^20^ showed ICR changes in different vertebral movements, where ICR moved anteriorly with vertebral flexion, and posteriorly with vertebral extension. The normal localisations of ICRs according to age groups were defined by Liu *et al*^2^ at their large cohorts between 20-79 age patients. In the asymptomatic population, ICR was localised to the upper and posterior part of the lower vertebra at the associated vertebral segments. With aging, ICR moved into more anterior and superior location due to disc space degeneration, anterior osteophytes, and sclerosis.

Although primary outcome variable was ICR change we also evaluated the sagittal balance parameters in our study. Zhao *et al*^21^ conducted a study with 68 AIS patients, among which they compared T2, T3 and T4 levels, and found that upper instrumentation level was not effective in terms of change in cervical lordosis. However, Ketenci *et al*^22^ findings were different as they declared T2 and T3 instrumentation caused a decrease in cervical lordosis compared to the lower levels. They attributed this result to a decrease in T1S and T1-T5 kyphosis after T2-T3 instrumentation. We similarly found in our study as T2 instrumentation cause decrease in lower cervical lordosis. However, in our cohort neither T1 slope nor T1-T5 kyphosis were different between patients instrumented up to T2 or not. Smith *et al*^14^ showed that T1S was correlated with cervical lordosis and cervical lordosis was a compensation tool for positive sagittal balance to maintain horizontal gaze. Similar to this result, we found that T1S was correlated with LCL and SVA.

It is known that thoracic alignment is an important part of global sagittal alignment. The T6-T12 thoracic vertebras are responsible for 10% of cervical movement^23^. It is expected that cervical lordosis will improve after correction of thoracic hypokyphosis. However, Canavase *et al*^24^ found that cervical alignment was not affected by thoracic kyphosis or upper instrumentation level. In contrast, we found that postoperative lower cervical lordosis increased in patients with T5-T12 hypokyphosis (TK <20°) and decreased in patients with T2 instrumentation. Similar to our results, Hilibrand *et al*^25^ reported post-operative increase in cervical kyphosis in normokyphotic or hyperkyphotic patients, where a lordotic improvement was seen in hypokyphotic group. In another study, patients with acute neck pain, chronic neck pain and asymptomatic individuals were compared for cervical alignment and no statistically significant difference was found for ‘straight cervical spine’^26^. The authors concluded that the straight cervical spine does not reflect the muscle spasm caused by pain in the neck which supports our finding of cervical lordosis is a radiological instant view of the spine rather than clinical importance. In a recently purposed classification, Ito *et al*^27^ divided AIS population according to their cervical lordosis (>4°), cervical kyphosis (CK) (<-4°) and sigmoid (one segment kyphotic and one segment lordotic) cervical alignment. They further divided CK group into three subgroups: CK-H (Thoracic kyphosis (TK) apex is above T4 level), CK-M (TK apex is between T4-T9) and CK-L (TK apex is below T9 level). They claimed that CK H group is a compensatory cervical kyphosis for the relatively hypokyphotic thoracic region. Similarly, we figured out that [although not statistically significant but close (p: 0.056 r=0.387)] a decrease in T5-T12 kyphosis accompanied by LCL decrease (kyphotic vector).

This study has some limitations. First, this study tends to have a Type 2 error. Due to our relatively small cohort caused mainly by prospective nature of data collection, it is always hard to give a statistically significant result. Nevertheless, this was the first pilot study that utilises ICR for kinematic evaluation of cervical region after thoracic instrumentation with a relatively quiet number of patients. Secondly, the detection of ICR prone to have measurement error and sensitivity problem on behalf of the used technique. To minimise this, we utilised two software that would enable us to do more precise measurements compared to previous literature. Third, it would be better if we included clinical parameters such as Oswestry Disability Index, SF36 or Visual Analogue Scale at the start of data collection, which would give more information for the functional perspective of instrumentation. We used the measurement techniques from the published studies^2,17^. However, the evaluation of patients with their capability to do cervical flexion and extension was also subjective. Anyhow, this was a real-life data, and we urged the patients to reach their extension and flexion limits during image acquisition which shows their real activity level in daily life. Also, in terms of Lenke classification, the cohort composed of heterogenous population which might create confounding.

## Conclusion

The post-operative cervical lordosis or kyphosis view would not give an impression about post-operative cervical spine motion quality after thoracic instrumentation in AIS population. It is possible to see a kyphotic cervical spine with a well-established ROM or lordotic cervical spine with a decreased ROM. Post-operatively we detected a decrease in thoracic kyphosis and increase in TPA, SS, and SVA in a positive global balance trend. T1S plays a keystone role as it is correlated with every segment of sagittal spine such as LCL, T5-T12K, SVA, and C2-C7 SVA. We find out that patients with pre-operative thoracic hypokyphosis tend to gain more cervical lordosis post-operatively. In addition, T2 instrumented groups tend to lose their cervical lordosis.
